# Understanding the illicit drug distribution in England: a data-centric approach to the County Lines Model

**DOI:** 10.1098/rsos.221297

**Published:** 2023-05-03

**Authors:** Leonardo Castro-Gonzalez

**Affiliations:** ^1^ Department of Economics, University of Birmingham, Edgbaston, Birmingham B15 2TT, UK; ^2^ Public Policy Programme, The Alan Turing Institute, British Library, 96 Euston Road, London NW1 2DB, UK

**Keywords:** behavioural sciences, data sciences, spatial analysis, crime detection, County Lines Model

## Abstract

The County Lines Model (CLM) is a relatively new illicit drugs distribution method found in Great Britain. The CLM has brought modern slavery and public health issues, while challenging the law-enforcement capacity to act, as coordination between different local police forces is necessary. Our objective is to understand the territorial logic behind the line operators when establishing a connection between two places. We use three different spatial models (gravity, radiation and retail models), as each one of them understands flow from place *i* to *j* in a different way. Using public data from the Metropolitan Police of London, we train and cross-validate the models to understand which of the different physical and socio-demographic variables are considered when establishing a connection. We analyse hospital admissions by drugs, disposable household income, police presence and knife crime events, in addition to the population of a particular place and the distance and travel times between two different locations. Our results show that knife crime events and hospital admissions by misuse of drugs are the most important variables. We also find that London operators distribute to the territory known as the ‘south’ of England, as negligible presence of them is observed outside of it.

## 1. Introduction

Over the last decade, a new illicit drugs distribution model has been developed in the UK. The model was baptized as the ‘County Lines Model’ (CLM) by the UK Government [1] given its use of phone lines to connect clients and sellers residing in different counties.

The problem has become increasingly worrying each year, becoming a top priority for security agencies given their limited ability to stop them, the modern slavery practices and the public health problems that the CLM brings to local communities [1–7].

The *modus operandi* can be described in the following way: a central hub is settled in big English cities like London, Birmingham, Manchester or Liverpool, from where drugs are sold and distributed [8]. From these hubs, *lines* are settled in other parts of the country where a local market is established. So-called *settlers*, find a local accommodation (normally a flat belonging to consumers) in the destination market from which drugs can be distributed. Local *runners* are then hired to distribute the illicit merchandise to the consumers. Runners tend to be young people with knowledge of the local market whose tasks are to deliver merchandise and attract new clients. The distribution model increases the efficiency with respect to ‘the traditional model’ [8] where the ‘highstreet’ illicit drug seller buys merchandise from a bigger distributor, to then sell it on the street. The improvement of the CLM is to merge both tiers (local and bigger seller) uniting both channels of distribution (hub-settler and runner-consumer).

Local consumers are given a phone number where they can place an order. The call is normally picked up in the central hub, from where they make the arrangements to distribute it to the consumer via the settler and the runner. The settler travels back and forth from the central hub and the local market bringing merchandise, while the runner distributes to the final consumer.

According to the National County Lines Coordination Centre [9], three cities account for more than 80% of the detected county lines in Great Britain in 2019 and 2020. These are, in respective importance, London, Birmingham and Liverpool. Public data are scarce, only having detailed records for London for those 2 years.

The implications of the proliferation of the CLM in the UK are multiple. Three are particularly highlighted in the literature [3,4,10]: (i) the rising of new illicit drug markets in small coastal towns and rural areas of England where illicit drug problems were not found before; (ii) also, the involvement of young and vulnerable people in the distribution scheme is a cause of concern for the UK Government. This population is the most prone to be caught by law-enforcement bodies, while also being involved in a modern slavery scheme making them hard to leave the CLM once they are involved; and (iii) finally, a limited ability of the different police forces in England to dismantle any complete distribution channel between one place and another. Cooperation between different law-enforcement bodies is necessary, as every link in the distribution engine can work autonomously, making it hard for bodies to dismantle the whole distribution operation.

The fact that county lines operators are found in small villages and coastal towns, far from local capitals and larger cities has given rise to different hypotheses about the logic behind establishing a *line*. Indeed, the population of a given place seems not to be an essential element to establish a local market, as large population centres (London, Manchester, etc.) do not attract a big number of lines according to public data shown in the strategic report from the National County Lines Council [9]. According to the same report, the logic behind the gangs operating county lines is a supply-demand balance.

The main objective of this work is to better understand the *territorial logic* that county lines operators follow to establish different distribution routes. To do so, we have to answer the question if the ‘traditional distribution model’ has been broken as the literature suggests [5]. The traditional model refers to what has been seen in the UK for decades where a ‘highstreet dealer’ would buy merchandise from bigger distributors to then sell it in their local market [8]. If so, which are the new social, demographic and economic elements that are now taken into account to establish a new route? To answer both questions would help to obtain useful information for the Metropolitan Police to understand and tackle the county lines problem.

We test three different spatial interaction models to compare flows from one place *i* to a second place *j*. We understand each of these models as different ways to understand the flow of persons/merchandise. Thus, by testing and comparing them we can extract information about which underlying mechanisms could county lines operators follow. The models we use are the Gravity Model [11], the Radiation Model [12] and the Retail Model [13], taking a similar approach as in [14] while extending it and adapting it to accomplish our objectives.

We use the classic Gravity Model as our benchmark, as it understands the flow from one place to another as proportional to the respective populations and inversely proportional to the distance between both places. Thus, we use it as a proxy of the traditional idea stating that more population would translate into more demand for illicit drugs. In that sense, a more populated city like Birmingham or Manchester would be more attractive for county lines operators than other rural places at the same respective distances from a given origin.

The Radiation Model understands flows as a process of sorting the available opportunities between *i* and *j*. To arrive at place *j*, the studied element (person/merchandise) should not be captured by the opportunities found on the way to it. In this case, we are testing the *distribution of population* in England. That is, for example, not only taking into account the population of Birmingham and London, but also the population found in between.

Finally, the Retail Model understands flow as a balance between the opportunities and the costs of going from one place to another, compared with all the other competing places in the given space. This latter model allows us to test another kind of dynamics involving different benefits and costs while considering competition too. The different benefits and costs can be of physical nature (time, distance), but also social or demographic. We explore five different independent variables we expect to have some leverage for operators. These are knife crime events, number of police officers, gross disposable household income and hospital admissions by misuse and poisoning by drugs as possible costs. The five variables are considered *per capita*, and in the case of hospital admissions, by hospital bed too.

The hospital admissions are taken as proxies for illicit drugs consumption, as no other data is available. In that sense, we are exploring correlations between other social elements that might be of higher importance for county lines operators to establish local markets. Knife crime events are another high-priority incidents for the UK Government [15] which are reported to be related to gang rivalry. We are interested to see if the presence of these events could be considered a disincentive for establishing a local market. In the same way, we are expecting the police workforce to be a disincentive for gangs. Finally, we take the gross disposable household income as a measure of richness, as average income does not take into account regional disparities in rent prices, money transfers from the government and local taxes. We train and test the three models with public data from the Metropolitan Police of London [16,17], accounting for the detected lines in other police force territories in Great Britain from London in 2019 and 2020.

The information that the Metropolitan Police gives around the reported number of county lines in [16,17] is not enough to know if any variation between one year or another is because of particular changes enforced by the Police, or any other causal element. Data thus remains aggregated over time, only observing the final balance (new detections minus lines taken down).

The point mentioned above also raises an important issue about how this work should be interpreted. Given the granularity of the Metropolitan Police data, we cannot model individual decisions. Thus, the studied variables must be taken as associated with other unobservable confounders that the operators take into account, rather than variables the operators directly consider to establish a market.

In the following, we present a brief literature review in §1.1. We also present the different models and the data tested in §2. Results are presented in §3, followed by a discussion and a conclusion in §4. In the latter section we also discuss the limitations of this work. We also present two appendices in the form of the electronic supplementary material; appendix S1 is a table that assists the reader with the models tested, while appendix S2 details the different sources and formats of the data used in this work. To the authors’ knowledge, this is the first published work that quantitatively examines the CLM.

### 1.1. Related work

In the case of the CLM, only qualitative and official literature has been published. The official literature includes documents and reports from different police agencies and the UK Government. In particular, the National Crime Agency (NCA) has published each year a statement regarding the views of the organization about the CLM [1]. The document presents the findings from the NCA to understand the model and the different consequences it has had on the population.

In 2019, the UK Government’s Home Office commissioned an up-to-date report to be done around the illicit drugs problem in the UK. The report was published in early 2020 [2,10] and reveals how the CLM has evolved over the last decade. It also reports how the consumption of illicit drugs has changed in the population, stating that the UK faces an important challenge, as there currently exist two peaks of consumers: one in their 20s and another in their 60s. Each one of those is increasingly worrisome, as the first one is the future workforce of the UK and the second represents an increasing pressure on the public services.

Two different police organizations have published information about the CLM information they have. These are the Metropolitan Police of London [16,17] and the West Midlands Police (Birmingham and metropolitan area) [18]. Only the Metropolitan Police has published quantitative data about their detection of lines in other police territories.

In January 2018, a debate was held in the House of Commons (UK’s lower parliamentary chamber) to discuss the exploitation and harm done by the CLM in London [19]. Different members of the Parliament asked what has been done until that point to tackle the CLM problems in London, particularly gang activity and exploitation.

Outside official documentation, academic literature about county lines has mostly been dedicated to report child exploitation in different locations of England [3–5] and Scotland [20]. In all of them, we find a description of the model. An anthropological study can be found in [8], where the authors interview different consumers and victims of the CLM in south England.

The present research is also found in the current context of the need for better information for law-enforcement bodies in the UK, as there is an ongoing discussion about how Brexit and the COVID-19 pandemic will have a major effect on public spending, particularly in law-enforcement bodies and the National Health Service (NHS; the public health body in the UK) [21]. In particular, reports state historical maximum numbers of drug-related deaths *per capita*, as a new generation of young consumers enters the market and an older generation requires more healthcare services [2]. Also, it has been discussed how Brexit would make it more difficult for the United Kingdom to access and profit from European funding and infrastructure (like the European Monitoring Centre for Drugs and Drugs Addiction, EMCDDA) for better intelligence and tackling strategies for a better public health and general quality of life for its citizens [22].

## 2. Methods

We cannot speak of a flow of persons, but rather a number of detected *lines* (connections) established from a place *i* to another place *j*. In that sense, a data point is a natural number, $T_{ij}^{\mathrm{data}}$, representing the detected connections.

The spatial resolution we work with is at police force territory, which in Great Britain accounts for 39 in England, five in Wales and one in Scotland. In our case, we work with the 39 territories in England only to train our models. We only train for England as not all features used in the models are available for the whole of Great Britain. We merge both territories in Greater London (Metropolitan Police + City of London Police) to work with London as a unique space.

### 2.1. Retail Model

The Retail Model was first presented in [23] as an entropy-maximizing model for the function $T_{ij}^{\mathrm{retail}}$ with three different conditions: (i) an outflow condition $\sum_{\;j}T_{ij}^{\mathrm{retail}}=T_{i}$; (ii) a Boltzmann-inspired energy conservation condition with respect to the travel time *c*_*ij*_ from *i* to *j*, $\sum_{i,j}T_{ij}^{\mathrm{retail}}c_{ij}=C$; and (iii) a similar conservation condition with respect to the total *benefit* found in the space, $\sum_{ij}T_{ij}^{\mathrm{retail}}\log w_{\;j}=B$, where *w*_*j*_ is the benefit of place *j* to attract people.

Using the maximum entropy principle with the three constraints described above, we obtain the resulting function for $T_{ij}^{\mathrm{retail}}$:

2.1 $$T_{ij}^{\mathrm{retail}}=\frac{T_{i}\exp\{\alpha \log w_{\;j}-\beta c_{ij}\}}{\sum_{k}\exp\{\alpha \log w_{k}-\beta c_{ik}\}},$$

where *α* and *β* are two free parameters coming from the maximum entropy derivation. Notice how the exponent in the numerator represents the balance from the benefits at *j* and the cost to get to *j* from *i*, given by *α* log *w*_*j*_ − *βc*_*ij*_. This latter balance competes with the other balances of going to the places *k* via the denominator of equation (2.1).

The retail system has been studied for different spatial dynamics in the past [14,24], allowing for inclusion of different types of data as benefit *w*_*j*_. In this case, as we are interested in knowing if different social variables (police workforce, knife crime events, hospital admissions by misuse of or poisoning by drugs and drug-related deaths) might be relevant benefits or costs for the county lines operator, we thus replace condition (iii) mentioned above by five analogous restrictions, one per variable, and use the different $w_{\;j}^{\left(n\right)}$ as the social/demographic variables. All of them (gross disposable household income, police workforce, knife crime events and hospital admissions) are normalized by the population of the police territory so they become per 100 000 inhabitants. We thus obtain as the final solution:

2.2 $$T_{ij}^{\mathrm{retail}}=\frac{T_{i}\exp\{\sum_{n}\alpha_{n}\log w_{\;j}^{\left(n\right)}-\beta c_{ij}\}}{\sum_{k}\exp\{\sum_{n}\alpha_{n}\log w_{k}^{\left(n\right)}-\beta c_{ik}\}}.$$

By exploring the magnitude and sign of the different *α*_*n*_, we can then have an insight about the elements that correlate to the detected lines from the Metropolitan Police, and if the variable is perceived as a benefit (*α*_*n*_ > 0) or a cost (*α*_*n*_ < 0).

### 2.2. Gravity Model

The Gravity Model computes flows from *i* to *j* as proportional to the product of populations of *i* and *j*, and inversely proportional to the distance between them. The model has different expressions and different limitations [12,25]. We take as a basis for this work the following form [11]:

2.3 $$T_{ij}^{\mathrm{gravity}}=G\frac{m_{i}^{a}m_{\;j}^{b}}{d_{ij}^{c}}.$$

We impose the outflow restriction $\sum_{\;j}T_{ij}^{\mathrm{gravity}}=T_{i}$, which makes equation (2.3) become

2.4 $$T_{ij}^{\mathrm{gravity}}=T_{i}{(\sum_{k\neq i}\frac{m_{k}^{b}}{d_{ik}^{c}})}^{-1}\frac{m_{\;j}^{b}}{d_{ij}^{c}}.$$

### 2.3. Radiation Model

The idea behind the Radiation Model originally comes from a particle transmission and absorption model in physics, where a particle is supposed to be emitted from place *i* and arriving at place *j* by sorting all *opportunities* in the way, i.e. not being absorbed on the way from one place to another. This idea has been applied to the flow of persons in a given space, first used as a commuter model for job seeking in the United States [12], to then being applied to different examples where commuters are modelled [26,27]. The original formulation of the radiation model is

2.5 $$T_{ij}^{\mathrm{rad}}=T_{i}\frac{\;p_{i}p_{\;j}}{\left(p_{\;j}+p_{ij})(p_{i}+p_{\;j}+p_{ij}\right)},$$

where *p*_*i*_ and *p*_*j*_ are the populations of *i* and *j*, *p*_*ij*_ is the sum of populations between both places and *T*_*i*_ is given by the outflow constraint $T_{i}=\sum_{\;j\neq i}T_{ij}^{\mathrm{rad}}$. In this particular project we work with a modified version from [28]:

2.6 $$T_{ij}^{\mathrm{rad}}=T_{i}\frac{P(1|n_{i},n_{\;j},n_{ij})}{\sum_{k}P(1|n_{i},n_{k},n_{ij})},$$

where *n*_*i*_, *n*_*j*_ and *n*_*ij*_ are the opportunities in *i*, *j*, and between both places, respectively. In this case, we simply suppose that *n*_*i*_ = *ρp*_*i*_, with *P*(1|*n*_*i*_, *n*_*j*_, *n*_*ij*_) as the probability of the ‘particle’ being absorbed on the way from *i* to *j* given the opportunities *n*_*i*_, *n*_*j*_ and *n*_*ij*_:

2.7 $$P(1|n_{i},\;n_{\;j},\;n_{ij})=\frac{\left[{\left(n_{i}+n_{\;j}+n_{ij}\right)}^{r}-{\left(n_{i}+n_{ij}\right)}^{r}](n_{i}^{r}+1\right)}{\left[{\left(n_{i}+n_{ij}\right)}^{r}+1][{\left(n_{i}+n_{\;j}+n_{ij}\right)}^{r}+1\right]}.$$

### 2.4. Model selection process

The three models presented above represent different spatial interactions, interpreted in this context as different decision processes from the county lines operators to establish a connection between places *i* and *j*. To compare the different models and select the most appropriate one for our available data, we proceed using two different measures found in the literature: the Sørensen–Dice index *S* [26], and the Bayesian information criterion (BIC) which is based in the maximum-likelihood principle [29].

The Sørensen–Dice *S* index measures the similarity between two different samples. Given a modelled number of detected lines $T_{ij}^{\mathrm{model}}$ after any of the models described above, and the observed data $T_{ij}^{\mathrm{data}}$, we use the same formulation as in [26]:

2.8 $$S=\frac{2\sum_{i,j}\min(T_{ij}^{\mathrm{data}},T_{ij}^{\mathrm{model}})}{\sum_{i,j}T_{ij}^{\mathrm{data}}+\sum_{i,j}T_{ij}^{\mathrm{model}}}.$$

We perform a twofold cross-validation, splitting our database for 2019 and 2020. Thus, training with 2019 (2020) data to then validate with 2020 (2019) data. The main argument around why we perform a twofold cross-validation, and not an *n*-fold one with a higher *n* is that, in order to comply with an accurate comparison between the different models, the cross-validation must be performed in the same folds for each of the models. By including the radiation model in equation (2.6) which works in slices of land rather than individual points, we would then have to correctly choose our different folds, so no information is lost when slicing. However, given the topology of England and the way the variable *n*_*ij*_ is constructed for equation (2.6), we could only slice England in two different pieces, which by themselves are not well balanced (the southeast of England, and the rest of the country).

As an extra criterion to model selection, we also compute the BIC to the whole modelled sample by each of the models. BIC computes the log-likelihood and corrects it with the size of the sample *M* for each model. In that sense,

2.9 $$\mathrm{BIC}=2\log M-2\log\hat{L}.$$

*M* is the size of the sample and $\log\hat{L}$ represents the maximum value obtained for the log-likelihood when training the model. The log-likelihood is computed with the parameters that minimize the loss functions used to calibrate the model.

As discussed before, given the nature of the detected lines by the Metropolitan Police, we are interested in testing two different loss functions: the usual mean-square loss function derived from a Gaussian likelihood, shown in equation (2.10), and a loss function derived from a Poissonian likelihood, shown in equation (2.11). The choice of the Poissonian likelihood is given by the distribution of lines detected for both years, while the mean-square loss function is chosen to be a benchmark with respect to equation (2.11):

2.10 $$L_{G}({\left\{T_{Lj}^{\mathrm{model}}(\hat{\;\theta })\right\}}_{\;j}\;|\;\hat{\;\theta })=\frac{1}{2N}\sum_{\;j}{(T_{Lj}^{\mathrm{data}}-T_{Lj}^{\mathrm{model}})}^{2}$$

and

2.11 $$L_{P}({\left\{T_{Lj}^{\mathrm{model}}(\hat{\;\theta })\right\}}_{j}\;|\;\hat{\;\theta })=\frac{1}{N}\sum_{\;j}T_{Lj}^{\mathrm{model}}-T_{Lj}^{\mathrm{data}}\log T_{Lj}^{\mathrm{model}},$$

where $\hat{\theta }$ is the vector of free parameters for each model. To each of both loss functions, we are also adding an L2 regularization term $\lambda \|\hat{\theta }{\|}^{2}$, with *λ* = 1. The subscript L in *T*_*Lj*_ represents London, thus showing the observation/model for London to any other police territory *j*.

### 2.5. Pipeline

The analysis pipeline is as follows: we perform a twofold cross-validation on each of the three types of models (Gravity, Radiation and Retail). In total, we are training one Gravity Model, one Radiation Model and 32 Retail Models. The 32 Retail Models are a result of adding an offset to the five different free parameters {*α*_*n*_} included in the Retail Model of equation (2.2). Thus, the total number of models is $\sum_{i=0}^{5}\left(\begin{array}{c} 5\\ 1 \end{array}\right)=32$. For all the 32 models we still take into account the *β* parameter which accounts for the travel times cost. A more detailed list of the models trained can be found in the electronic supplementary material, appendix S1. The models are trained using two different cost functions described in §2.4, and evaluated using the Sørensen–Dice index [26] and the BIC [29].

### 2.6. Data

In this section, we describe the different data that is implemented in the different tested models. In the electronic supplementary material, appendix S2 we offer a more detailed description of the complete database used. The three models (Gravity, Radial and Retail) have as one of the inputs the population of the police territories (directly or indirectly). These are public data from the Office of National Statistics (ONS), and by the time of submission, the last published update is of 2019.^1^

The Gravity Model and the Retail Model, respectively, use the distance and the travel time from one place to another. Given that the used resolution is at the police territory level, we are using the distance/travel time from the most populous place in territory *i* to the most populous place in *j*. Data are drawn using the Google Maps © API.

The exponent of equation (2.2) allows us to compute a balance between the different benefits and costs of going from *i* to *j*. The training and comparison process taken in this work allows us to know if a given variable is a cost or a benefit, thus allowing testing between different variables.

An important feature to test is the number of potential customers for the county lines operators. This accounts for current and potential consumers. We use two different measures as a proxy for this consumption: finished hospital admissions [30] by misuse of drugs and finished admissions by poisoning of drugs. Hospital admissions are normalized by population and by daytime hospital beds *per capita*.

Another feature we test is the police workforce in each territory. We use the number of average full-time police officers over the British fiscal year (May–April) which can be obtained from Flatley [31].

To account for the disparities of richness in the different parts of England, we use the gross dispensable household income (gdhi). In comparison with the household income, the gdhi takes into account the amount of money that households have after local and national income taxes and benefits from the government. Data were obtained from the ONS [32].

Finally, we are interested in testing the knife crime events *per capita* in each of the police territories. Knife crime events have been an increasingly worrying matter for the British Government, with numbers increasing 78% in England from 2014 to 2020 [15].

## 3. Results

### 3.1. Model selection

**Figure 1.**
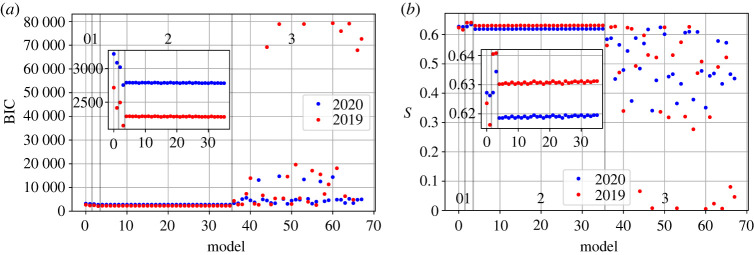
Results of BIC and the Sørensen–Dice index *S* for the 68 different models tested. Zone 0 corresponds to the Gravity Model. Zone 1 corresponds to the Radiation Model. Finally, zones 2 and 3 correspond to the Retail Model with the Poissonian loss function and with the mean squared error (MSE) loss function, respectively. The annotated year corresponds to the data on which the model was trained on. Details of each model can be found in the electronic supplementary material, appendix S1.

Results for the BIC and the Sørensen–Dice index are found in figure 1*a* and in figure 1*b*, respectively.

When comparing the Retail Model calibrated with a Gaussian loss function (zone 3 in figure 1) with respect to the other models, we observe how it performs worst in all of its forms for both the BIC and the *S* index. We can thus proceed to discard this zone.

**Figure 2.**
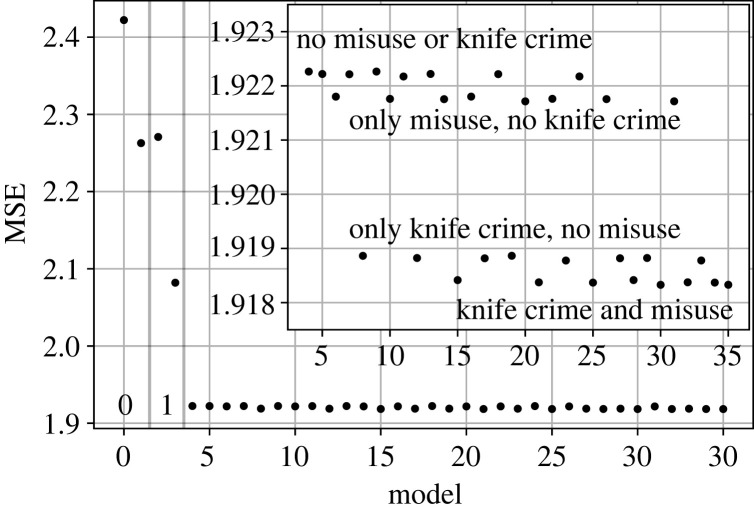
MSE costs when comparing the trained models with the Metropolitan Police data.

With the models left, we perform a comparison by computing the mean squared error (MSE) between the Metropolitan Police data for both years (2019 and 2020) and the predictions obtained from each model. The MSE is computed with the logarithms of the data points, so in this case $\mathrm{MSE}=(1/N)\sum_{i,j}{\left(\log T_{ij}^{\mathrm{data}}-\log T_{ij}^{\mathrm{model}}\right)}^{2}$, results are seen in figure 2.

The best-performing model is the Retail Model trained with the 2019 data and the Poissonian loss function. However, as it can be seen in the inset plot in figure 2, the results can be differentiated into four distinct levels. When examining each one of them, we observe how the hospital admissions by poisoning of drugs, the disposable income and the police presence variables do not have a significant effect on the performance of the model. This can be seen in the upper level, as those combinations not containing the knife crimes and hospital admissions by misuse of drugs variables are found there (all the different models are in the electronic supplementary material, appendix S1). The fact that the combination just considering the travel times is found there allows us to interpret that the three above-mentioned variables have a negligible effect on the performance of the model. The hospital admissions by misuse of drugs seem to have an impact on the cost, although not as important as the knife crime variable. When combining both variables we obtain the most important effect on the MSE cost and the best-performing models.

The Radiation Model follows as best performing when trained with the 2019 data and Poisson loss function. Finally, we obtained the Gravity Model trained in the same way.

In table 1, we detail all the selected models. To keep the selected models as simple as possible, we filter out all the different Retail models and keep only those with the minimum number of variables. That is, one with both the misuse and the knife crime variables in addition to the travel times, one with only the knife crime variable and travel times, one with only the misuse variable and travel times, and finally one with only travel times.

**Table 1.** Results for the best three models calibrated.

ranking	model	loss function	training year	parameters	BIC	*S*	MSE
1	Retail	Poisson	2019	*α*_2_ = −7.74 × 10^−3^,			
				*α*_4_ = −0.013,			
				*β* = 0.014	2280.0	0.6312	1.9184
2	Retail	Poisson	2019	*α*_4_ = −0.013,			
				*β* = 0.014	2282.5	0.6308	1.9189
3	Retail	Poisson	2019	*α*_2_ = −7.77 × 10^−3^,			
				*β* = 0.014	2286.8	0.6306	1.9218
4	Retail	Poisson	2019	*β* = 0.014	2288.8	0.6302	1.9223
5	Radiation	Poisson	2019	*ρ* = 2.085,			
				*n* = 1.038	2150.8	0.6408	2.0820
6	Gravity	Poisson	2019	*b* = 0.697,			
				*c* = 0.368	2414.5	0.6162	2.2628

From the exponent in equation (2.2), *α*_2_ corresponds to the hospital admissions by misuse of drugs, and *α*_4_ to the knife crime events. All variables are normalized by population.

We also select the best-performing Radiation and Gravity models as we are interested in comparing them with respect to the Retail Model.

As it can be seen from table 1, the two exponents *α*_*n*_ are negative, which is interpreted as the knife crime events *per capita* and hospital admissions by misuse *per capita* representing a cost to county lines operators. This will be discussed in §4.

### 3.2. Model analysis and geographical distribution

**Figure 3.**
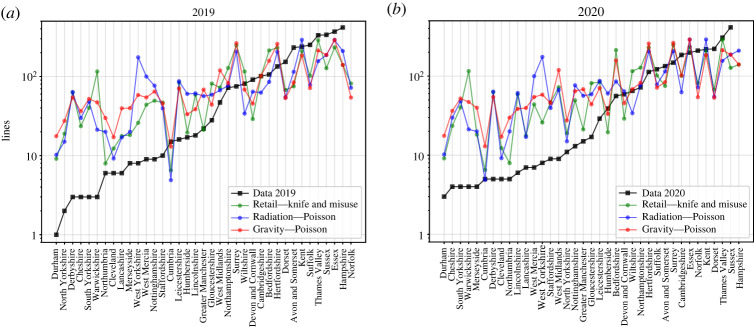
Data points and modelled lines ordered by police force for (*a*) 2019 and (*b*) 2020.

Once we have obtained the best-performing models, we proceed to compare and analyse their predicted lines. In figure 3, we present the different models compared with the Metropolitan Police Data for 2019 and 2020.

The three selected Retail models from table 1, which have the minimum number of variables, are depicted: one with both the misuse and the knife crime variables in addition to the travel times (model 1), one with only the knife crime variable and travel times (model 5) and one with only the misuse variable and travel times (model 6). These three models show very similar predictions and are, therefore, depicted together.

The three models tend to overestimate the detected connections to places with less than 70 lines, while tending to underestimate them in police territories with more than 100 lines detected.

Each one of the models has different ways of understanding the dispersion of flow in a given space. The calibrated Radiation Model views the flow from London to another given police territory as a process of sorting opportunities presented on the way. Opportunities are seen as proportional to the population by the value of *ρ* given in table 1. Thus, we are actually exploring how the population is distributed in England.

The Retail Model understands flow as a balance with respect to travel times and the other social variables using an exponential distribution. This means that flow from London to another police territory is given by how much time is spent commuting with respect to the other police territories and how much the other benefits/cost relate to it. Thus, a closer place from London would be favoured with respect to a farther one. However, given that this consideration is given by an exponential distribution, we can expect a slow decrease of lines when increasing travel times (light tail distribution).

Finally, the Gravity Model explores the flow with respect to the distance between two places and the population of the target place. In that sense, closer and more populous locations would take most of the outflow, while distant and less populated locations would be disfavoured by the model.

**Figure 4.**
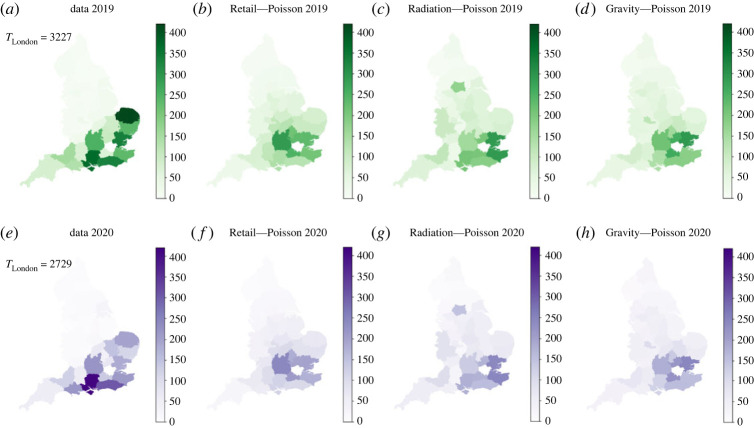
Heatmaps for the Metropolitan Police data for 2019 (*a*) and 2020 (*e*), and the three different models tested: Retail (*b*,*f*), Radiation (*c*,*g*) and Gravity (*d*,*h*).

**Figure 5.**
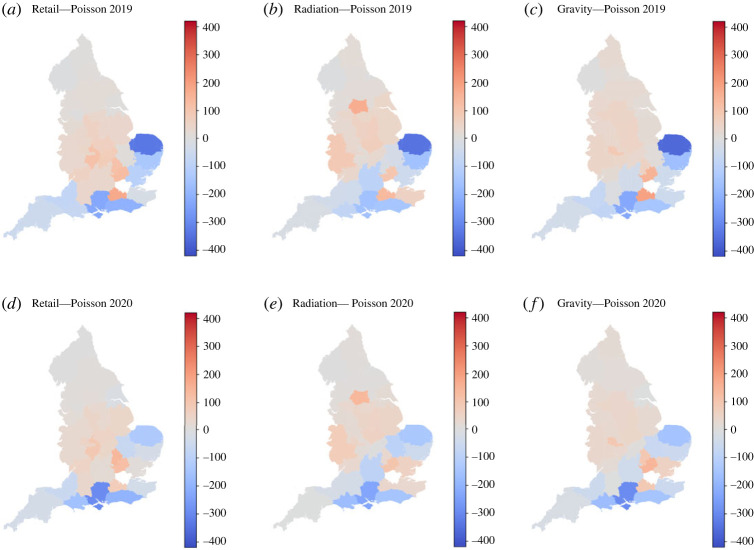
Heatmaps showing the difference between the modelled distribution of lines with respect to the Metropolitan Police data for 2019 and 2020.

To further understand the different results shown in table 1 and in figure 3, we map the different models and compare them with the Metropolitan Police data. This is shown in figure 4. While figure 4*a*,*e* present the Metropolitan Police data for 2019 and 2020, the rest present the modelled spatial distribution of lines. We also present the differences between the Metropolitan Police data and the models in figure 5. Red zones correspond to territories overestimated by the model, while blue zones correspond to territories underestimated by the model.

We start by analysing figure 4*a*,*e* corresponding to the Metropolitan Police data. The first thing to note is the decrease in detected lines in 2020 with respect to 2019. This effect can be given by mainly two factors taking into account the COVID-19 situation throughout 2020: the police having a smaller capacity to detect, or the reduced mobility in the country resulting in a decrease in the number of connections. However, the decrease is not generalized and we can observe an increase in some police territories from 2019 to 2020, as in Hampshire (south of England) where we find the maximum number for 2020.

An important second element to note from the ground truth data is a very high share of the total lines (94.02% for 2019 and 93.77% for 2020) concentrated in 16 out of the 37 police territories considered. This set of 16 police territories, in addition to London, is considered to be the ‘south’ of England, a social region with no administrative recognition which encloses the most developed parts of England and which opposes the ‘north’ of England, where more industrial cities like Manchester and Liverpool are found (for a study using percolation theory please refer to Arcaute *et al.* [33]).

The ‘north–south’ division is an element which none of the models captured. However, we can still see different ways of simulating the problem in figure 4. As discussed before, the Retail Model distributes the lines in what appears a concentric fashion with respect to London, leaning towards the centre of England. This can be seen more clearly in figure 5*a*,*d*, where we observe an overestimation in the Midlands and an underestimation of the coastal territories of the ‘south’. Note how the far southwest of England (Cornwall and Devon), which is farther away in travel times than the centre of England from London, is under-represented. This suggests that the operators in London would not have as primary factor for establishing connections the travel times to the different territories. This argument is supported by the opposite fact, where we observe an overestimation by the Retail Model in more connected places from London, like the West Midlands (Birmingham) and Warwickshire (south of Birmingham).

The Radiation Model understands the flow in a different fashion, as seen in figure 4*c*,*g*. In a similar way as the Retail Model, the ring surrounding London is still catching an important number of lines. However, we can also observe a number of relatively large hotspots, particularly in West Yorkshire (north of England) and in West Mercia (border with Wales). While the former territory includes important cities and urban centres such as Leeds and Bradford, West Mercia is a diverse territory with dense suburban counties belonging to the Birmingham metropolitan area and more rural areas towards Wales, like Shropshire. In figure 5*b*,*e* we observe also how the territories between West Yorkshire and London were filled with lines by the Radiation Model. It is also important to note how the big metropolitan areas in England such as Birmingham do not appear as hotspots in figure 4*c*,*g*.

Both models described above tend to distribute the number of lines in the centre of England, while avoiding the big cities. This is in contrast with the Gravity Model (figure 4*d*,*h*) where we observe the appearance of Birmingham and Manchester (second and third most populous cities in the UK) as county lines hotspots.

The three models do not detect the territories where the maximum number of lines are detected, like Norfolk in 2019 and Hampshire in 2020. On one hand, this is a sign of no overfitting from both models, but on the other hand, makes it very difficult for the models to detect future hotspots in the south of England.

## 4. Discussion

Our study focuses on the CLM distribution method of illicit drugs in England. We aim to investigate the territorial logic underlying the data accounting for the detected connections (lines) by the Metropolitan Police of London in other police territories [2,10,16,17].

We understand the number of detected lines as a flow of people/merchandise that starts in London and finishes in a given police territory. In that sense, by modelling the flow from one place to another and comparing it with the available data, we obtain information about which elements are present when establishing a local market.

Three different models are studied and compared. Each one of them follows, by construction, different logic about how to understand the flow from one place to another. The first one, the Gravity Model [11,25], sees flow as proportional to the population of both places, while inversely proportional to the distance between them. We take this model as our benchmark as it represents the classic idea that populous places would draw more attention than others given the same distance. The second, the Radiation Model [12], understands flow to a given place as a process of sorting the presented opportunities before arriving to the final destination. With this second model, we tested if the distribution of the population in England was involved in the decision-making process. Finally, the Retail Model [13,23] takes into account the balance between the benefits and the costs of establishing a market in a particular place with respect to all of the other possible places. This final model allows us to include as potential benefits/costs different social variables that we tested, like police workforce, knife crime events, hospital admissions for drug poisoning and for drug misuse.

We train the models using the Metropolitan Police of London data [16,17], and compare them using the BIC [29] and the Sørensen–Dice index [14] over a cross-validation. We also test two different loss functions, the classic MSE and the one derived from a Poissonian likelihood.

The best-performing model is the Retail Model, trained with the 2019 data, the Poisson loss function and with the hospital admissions by misuse of drugs *per capita* and knife crime events *per capita* as costs. In particular, knife crimes shows to be more important to hospital admissions when compared one to one.

The Radiation and Gravity models also perform correctly when trained with the 2019 data and the Poisson loss function. However, when comparing the predicted geographical distributions to the Metropolitan Police data, these two models predict hotspots in populous regions of England where no important number of lines were detected by the Metropolitan Police.

The fact that the Gravity Model did not perform at the same levels as the Retail or the Radiation models allows us to support the hypothesis in [8] stating that the ‘traditional model’ was broken. The original idea to include the Gravity Model with an outflow condition was to use it as a benchmark related to this traditional model to sell illicit drugs in large populous centres. Not being the best-performing model (and actually one of the worst-performing when using the Poisson loss function), we can then discard this territorial logic.

According to our ground truth, the distribution of the great majority of lines (93%) is over 16 of the 37 police territories in England, which form the union of the southwest, the southeast and the east of England. This territory is known as the ‘south’ of England.

While the Gravity and Radiation models overestimated different territories outside the south of England with large populations, the Retail Model did it in a more diffused way. This is owing to the exponential form of the model.

None of the three models could capture the hard border that the data shows between the south of England and the rest of the country. This raises the question about the characteristics of the 16 police territories that represent the ’south’ of England that make them so attractive for county lines operators. A first hypothesis is that the CLM, although not reported in the literature, actually acts within a more organized structure which can restrict itself to distribute in a given territory, as seen for other criminal organizations. In other words, even though not mentioned in the public information by the UK Government, different CLM gangs operating from London could restrict themselves to these 16 police territories as a measure to not enter into open conflict with other gangs from other CLM hubs. This hypothesis could be studied by having the data of the detected lines from the other important CLM hubs like Birmingham, Liverpool, Manchester and West Yorkshire. In that sense, we could expect a localized distribution in the ’north’ of England, obtaining a polycentric structure within the territory.

However, if the data from other CLM hubs would not comply with the segregation and rather concentrate on a subset of the 16 police territories considered as the ‘south’, then we would have a particularity of the consumers in those areas. This would also be of interest, as the population in this subset would have to have a distinction with respect to the other big metropolitan and rural areas of the 21 police territories left. This distinction, although it might be related to a particularity of the consumers, would also address the findings already obtained before in quantitative studies [33], where a clear distinction between the urban network between the south and the rest of England was found using percolation analysis.

The hypothesis about a polycentric structure could be supported by our findings on how knife crime events and hospital admissions by misuse of drugs are a cost for line operators. The fact that knife crime events appear as a cost might suggest an avoidance of certain gangs so conflict is spared. Hospital admissions, on the other hand, are used as a proxy to illicit drugs consumers given the lack of public information about it. In that sense, the fact that the hospital admissions variable is one of the two most influential variables, combined with the knife crime variable, could be interpreted as county line gangs avoiding places where there already is enough competition for them to handle. This competition can be regarded as a possible origin of conflicts (knife crimes) and responsible for having a greater share of the illicit drug consumption market in a given territory.

This comes to the main limitation of the present work: the amount of data and how the one available is constructed. The first point relates to how different interesting hypotheses that could help to understand the CLM at a larger scale cannot be tested. The second point relates to what the Metropolitan Police of London data represent. As it is an aggregated number of detected lines obtained from individuals having a relationship with the CLM [16,17], we can only suppose that this number is the total balance between those lines detected and those lines taken down by police enforcement (or any other exogenous cause). In the same way, with the reported data there is no information about any action that the different police bodies took to dismantle any reported line. Results then should be interpreted as associated with other unobservable confounders that operators consider to establish a local market.

We demonstrate that the territorial logic behind the CLM is not as simple as an offer–demand one, as different governmental institutions have published [1,2,9,10,16–18]. Instead, it might follow a social structure of the country, while also avoiding conflict with other gangs and markets already filled with competition. This by itself can be of great help for law-enforcement bodies, as it gives a good lead on where to look for the presence of county lines from London: places within the 16 police territories where there is not an important number of knife crimes *per capita*. There is no mention of these factors in the reviewed literature.

This work also allows us to implement a better coordination between local police forces, as the Metropolitan Police of London would only need to coordinate with 43% of the English police forces to tackle the 93% of the lines detected.

## Data Availability

All data and scripts regarding the analysis of this work can be found in the BritishDrugDynamics repository. The data are also provided in the electronic supplementary material [34].

## Footnotes

1 In general, all data obtained from the digital platforms of the British Government (gov.uk) is used under the Open Digital Licence.
